# A Vector Theory of Assessing Clinical Trials: An Application to Bioequivalence

**DOI:** 10.3390/jcdd11070185

**Published:** 2024-06-21

**Authors:** Vangelis D. Karalis

**Affiliations:** 1 Department of Pharmacy, School of Health Sciences, National and Kapodistrian University of Athens, 15784 Athens, Greece; vkaralis@pharm.uoa.gr; Tel.: +30-(210)-7274267; 2 Institute of Applied and Computational Mathematics, Foundation for Research and Technology Hellas (FORTH), 70013 Heraklion, Greece

**Keywords:** vector-based comparison, clinical trials, bioequivalence, endpoints, antihypertensives

## Abstract

A novel idea is introduced regarding the statistical comparisons of endpoints in clinical trials. Currently, the (dis)similarity of measured endpoints is not assessed. Instead, statistical analysis is directly applied, which can lead to multiplicity issues, reduced statistical power, and the recruitment of more subjects. The Vector-Based Comparison (VBC) approach originates from vector algebra and considers clinical endpoints as “vectors”. In the general case of N clinical endpoints, a Cartesian coordinate system is defined, and the most important primary endpoint (E1) is set. Following an explicitly defined procedure, the pairwise relationships of the remaining N-1 endpoints with E1 are estimated, and the N-1 endpoints are decomposed into axes perpendicular to E1. The angle between vectors provides insight into the level of dependency between variables. Vectors that are perpendicular to each other are considered independent, and only these are used in the statistical analysis. In this work, VBC is applied to bioequivalence studies of three anti-hypertensive drugs: amlodipine, irbesartan, and hydrochlorothiazide. The results suggest that VBC is a reproducible, easily applicable method allowing for the discrimination and utilization of the endpoint component expressing different attributes. All clinical characteristics are assessed with increased statistical power, without inflation of type I error.

## 1. Introduction

Clinical trials serve as the gold standard of experiments in the medical field, strictly designed to validate the safety, efficacy, and potential benefits of various clinical interventions [1,2]. These trials are structured investigations conducted in carefully controlled settings. An essential aspect of conducting clinical trials is the thorough selection and definition of appropriate endpoints [3,4], set by researchers to evaluate the effectiveness, safety, and overall success of a medical treatment (e.g., administration of medicine). Primary endpoints typically represent the main outcomes of interest, while secondary endpoints provide additional insights into the intervention, albeit of lesser importance [5,6,7,8]. Examples of endpoints used to express the antihypertensive effect in cardiology clinical studies include changes in blood pressure, percentage of patients achieving target blood pressure, and time taken to achieve target blood pressure.

Selecting appropriate endpoints requires careful consideration of various factors, including the characteristics of the disease or condition. It is crucial to choose endpoints that are clinically meaningful, reliable, and sensitive to changes induced by the treatment [3,4]. While some clinical studies may focus on a single primary endpoint, many trials incorporate multiple endpoints to provide a comprehensive assessment of treatment impact, encompassing efficacy, safety, and patient-reported outcomes [9,10,11,12,13,14]; however, sometimes clinical endpoints are related to each other, particularly when measuring complex effects like the antihypertensive impact of medications. These interrelated endpoints can lead to certain drawbacks in clinical research such as the potential for redundancy, where multiple endpoints may measure similar aspects of the intervention impact, leading to unnecessary duplication of effort and resources. Additionally, interrelated endpoints can increase the complexity of data analysis and interpretation, requiring sophisticated statistical methods to account for correlations between outcomes [11,12].

Multiplicity, which refers to the inclusion of multiple endpoints in clinical trials, can present several challenges such as the increased risk of false-positive findings (type I error) since conducting multiple statistical tests raises the probability of observing significant results by chance alone [15,16,17]; however, while avoiding multiplicity in clinical trials is important to minimize the risk of false-positive findings, it is also important to avoid the risk of increasing type II error (false negatives) [18,19,20,21,22]. The latter can arise from several factors like small sample size, inadequate study design, and insufficient statistical power. For example, as mentioned above, in assessing the efficacy of antihypertensive treatments, multiple related endpoints are often considered, such as the change in blood pressure, percentage of patients achieving target blood pressure, or time to achieve target blood pressure.

Similarly, in bioequivalence studies, which are used for the assessment of generics, multiple co-primary endpoints are used. Bioequivalence studies are clinical studies that are conducted in healthy volunteers to assess the pharmacokinetic similarity between the reference (R) and a test (T) pharmaceutical product. The area under the curve (AUC) is officially utilized to express the extent of absorption, while the maximum observed plasma concentration (Cmax) is used to express the rate of absorption [23,24]; however, emerging evidence, as well as previous observations, suggest that Cmax predominantly expresses the extent rather than the rate of absorption [25,26,27,28,29,30,31,32,33,34,35,36]. Quite recent machine learning studies have further demonstrated a high degree of correlation between AUC and Cmax, challenging the suitability of Cmax as a standalone endpoint for assessing absorption rates [37,38,39]. These examples underline the importance of reconsidering endpoint selection in clinical (and bioequivalence) studies to ensure accurate and meaningful assessments of the desired characteristics.

Thinking outside the clinical trials frame, the concept of comparing endpoints to quantify characteristics extends beyond and finds application in various scientific fields. In this context, clinical endpoints can be alternatively viewed as “vectors”, which allows the application of mathematical methods for analysis. In this vein, in vector algebra and in physics, vectors (of variables) can be related or independent, according to the angle between them [40,41]. Related vectors are those that can be expressed in terms of each other or are dependent on each other in some manner. On the other hand, independent vectors are those not directly linked to each other, representing distinct physical quantities or directions [40,41]. In addition, modern dimension reduction techniques allow for identifying relationships and patterns within endpoint data [42,43,44]; however, dimension reduction techniques, while powerful, face several challenges that can affect their applicability and reproducibility.

The aim of this study is to introduce a novel approach for evaluating clinical trials with multiple endpoints, considering not only their numerical values but also their physical properties and interrelationships. Traditional statistical assessments do not take into consideration the (dis)similarity of measured endpoints [6,7,8,22,23]; instead, statistical analysis is directly applied, which can lead to multiplicity issues, reduced statistical power, and recruitment of more subjects if these endpoints are interrelated. To address this, the new approach, termed “Vector-Based Comparison” (VBC), is inspired by dimension reduction and vector algebra principles and considers clinical endpoints as “vectors”. The VBC approach allows for the discrimination and utilization only of the endpoint component expressing different attributes; thus, this allows for the assessment of all different clinical characteristics with increased statistical power and no inflation of type I error.

Using bioequivalence studies as case studies, the application of the VBC method is demonstrated to datasets from three cardiovascular drugs (amlodipine, irbesartan, and hydrochlorothiazide), each with distinct absorption characteristics. Initially, principal component analysis (PCA) is used to uncover the underlying relationships among the pharmacokinetic endpoints. Subsequently, Monte Carlo simulations focusing on 2 × 2 crossover bioequivalence studies are conducted to implement the VBC approach and compare its effectiveness with the traditional method. It should be mentioned that the simulations performed in this analysis are only used as a means to demonstrate the appropriateness of the VBC method. In practice, simulations are unnecessary, and the application of VBC is easily applicable and fully reproducible, allowing for the exploitation of the full potential of endpoint data and providing a more comprehensive and insightful evaluation of clinical trial outcomes.

The subsequent sections of the manuscript are structured as follows: Section 2.1 provides a foundational overview of vector algebra, setting the stage for understanding the VBC theory elaborated in Section 2.2. The application of VBC in the context of bioequivalence is delineated in Section 2.3, while the technical aspects of principal components analysis and Monte Carlo simulations are elucidated in Section 2.4 and Section 2.5, respectively. Finally, the outcomes originating from the application of the VBC approach are detailed in the “Results” section.

## 2. Materials and Methods

### 2.1. Background Aspects: Endpoints and Statistical Assessment

**Figure 1:**
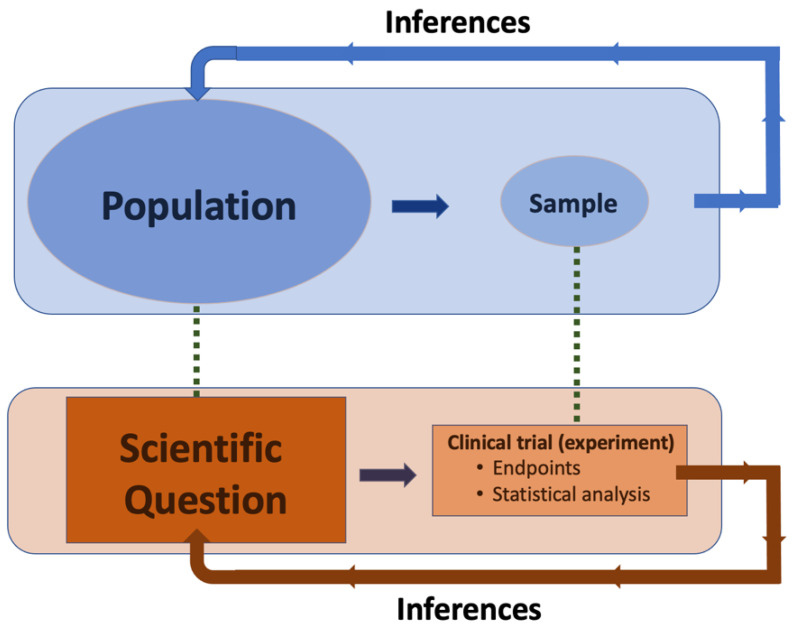
The basic dogma in statistics is that a random and representative sample of patients (or objects, etc.) is drawn from a population. The experiment (interventions) and statistical analysis occur within the sample, while the inferences made refer to the entire population from which the sample originates. Important aspects of the assessment include the application of the most appropriate statistical method and the selection of endpoints.

In general, the aim of clinical trials is to investigate the effect of an intervention/treatment (e.g., test medicine, T) against another intervention (e.g., the existing widely acknowledged intervention for example, the reference medicine, R). In order to accomplish this task, an appropriate and representative sample of volunteers (either patients or healthy subjects) is recruited [1,2,3,4]. In order to quantify the effect of each intervention, some measurable quantities are needed, the “endpoints”, which refer to specific outcomes measured to assess the effectiveness of a treatment or intervention [11,12]. After the endpoints are measured in each subject of the study, an appropriate statistical analysis follows that aims to identify whether significant differences exist between the groups under comparison. Based on the findings of the sample, inferences about the general population are made in order to answer the scientific question raised which was set a priori, namely in the protocol before initiation of the study. This is the basic dogma in every type of statistical assessment, including clinical trials (Figure 1).

It should be emphasized that critical aspects of the statistical process include [1,2,3,4]: (a) utilizing a representative sample; (b) ensuring an adequate sample size for sufficient statistical power; (c) measuring suitable endpoints to accurately express intended characteristics; (d) analyzing endpoints using appropriate statistical methods that meet assumptions and align with the aim of the comparison; and (e) ensuring the entire process is fully reproducible through clearly defined methodologies mandated by regulatory authorities and transparent, fully interpretable statistical methods.

The selection of endpoints in a clinical study requires careful consideration of various factors, to ensure that the study is scientifically sound, ethically conducted, and capable of providing meaningful insights into the effects of the intervention being studied [11,12,15,16]. In this context, a single or many endpoints are measured in a clinical study in order to express the desired characteristics. Even though these points can be suitable and important, however, there is nothing mentioned about the relationship among the endpoints [9]. Of course, the investigator does not intentionally include endpoints that express entirely the same characteristic but endpoints used to express a certain attribute may also inherently express other characteristics too. Thus, when these endpoints are undergoing statistical analysis, unavoidably we merely analyze the same attribute more than once. Nevertheless, no measures are taken either to exploit the similarity between the endpoints or, alternatively, to avoid including all of them. It should be mentioned that we are not referring to multiplicity issues; rather, the focus is on the direct or underlying relationships between two or more endpoints.

For example, for a clinical study measuring the antihypertensive effect of a drug, possible endpoints that could be used are changes in systolic and/or diastolic blood pressure, change in ambulatory blood pressure, percentage of patients achieving target blood pressure, and time to achieve target blood pressure, etc. [1]. These endpoints are directly related as they all measure the efficacy of antihypertensive drugs in reducing blood pressure. The percentage of patients achieving target blood pressure and the time taken to reach target levels are both influenced by the magnitude and speed of the reduction in blood pressure.

Another important aspect in clinical trials with multiple endpoints is multiplicity, which refers to the increased risk of false positives (i.e., type I errors) when multiple statistical tests are conducted simultaneously or sequentially within a study or analysis [18,19,20,21,22]. The issue of multiplicity is widely acknowledged, and suitable methods are commonly used to avoid it; however, until now, no measures have been taken to address endpoints that are related to one another, which is the focus of this study.

The purpose of this study is to first uncover this issue and then introduce a new approach to deal with it. The VBC idea proposed in this manuscript is explained in Section 2.2 and Section 2.3, while the technical aspects of its application in three cardiovascular drugs are presented in Section 2.4.

### 2.2. The Vector-Based Comparison (VBC)

The VBC idea originates from the recognition that endpoints are not only measurable quantities but can also be viewed as vectors. By conceptualizing them as vectors, it becomes possible to incorporate mathematical tools from other fields of science, such as vector algebra, into clinical trials.

#### 2.2.1. Elements of Vector Algebra

**Figure 2:**
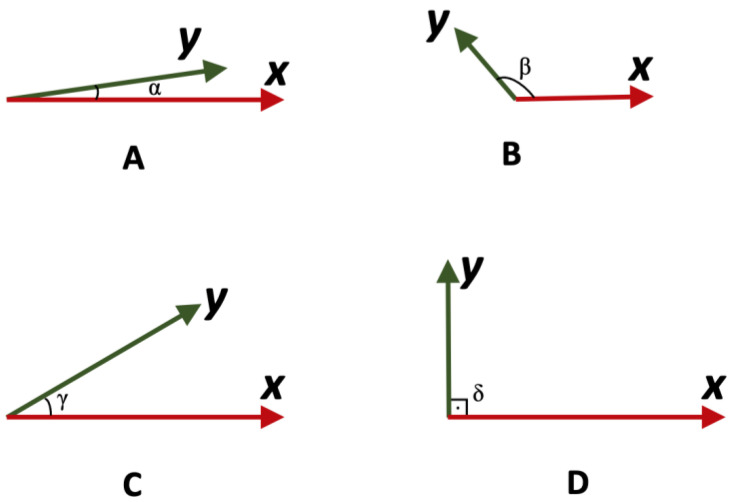
The relationship between two vectors, ***x*** and ***y***, is determined by the angle formed at the intersection of their tails. This angle can be acute (**A**,**C**), obtuse (**B**), or right (**D**), depending on the direction of the vectors.

Vector algebra is a mathematical discipline concerned with manipulating vectors, which are entities defined by both their size and direction [40,41]. Vectors are commonly represented geometrically as arrows in three-dimensional space, where the length of the arrow denotes the vector magnitude and its direction indicates the vector orientation. In this context, endpoints can be geometrically represented as vectors, and their comparison can be conducted similarly to the comparison of vectors. It is worth noting that the similarity between two vectors can be assessed using various methods, such as cosine similarity, dot (or “inner”) product, distances (usually the “Euclidean” or cosine distances), and correlation coefficient. In this context, the angle between vectors can provide insight into their similarity (Figure 2) [40,41].

Vectors that are closer in direction tend to have smaller angles between them (Figure 2A), indicating greater similarity. Conversely, vectors with larger angles between them are more dissimilar in direction (Figure 2B,C). Vectors that are perpendicular to each other (Figure 2D) are considered independent since a change in the horizontal component does not affect the vertical component. Every set that contains mutually perpendicular vectors is an independent set. In addition, when two given vectors are perpendicular then their cross product is not zero but the dot product is zero [40,41].

#### 2.2.2. Elements of Principal Component Analysis

The attributes mentioned in the previous section are in line with the terminology in dimension reduction techniques. For example, in PCA, two perpendicular vectors are considered independent of each other. PCA is a statistical technique used to simplify high-dimensional data by transforming it into a new coordinate system called principal components. These principal components are orthogonal to each other, meaning they are perpendicular vectors in the original data space [42,43,44]. Because perpendicular vectors in PCA represent different dimensions of the data and do not share any directional components, they are considered independent of each other.

#### 2.2.3. The VBC Concept Applied to Endpoints

**Figure 3:**
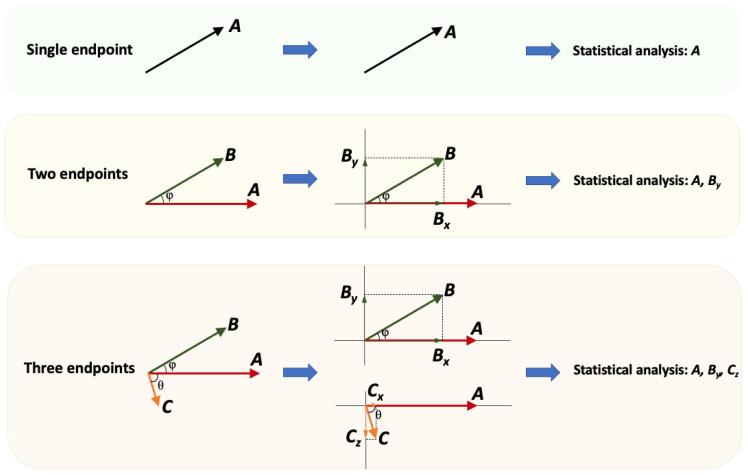
A schematic illustration demonstrating the decomposition of endpoints according to the VBC approach for clinical studies with multiple endpoints (two or more). The initial step involves defining the most significant primary endpoint (***A***). Subsequently, all other endpoints (***B*** and ***C***) are decomposed into a component that aligns with ***A*** and another component that is perpendicular to ***A***. Given that endpoints ***B*** and ***C*** represent distinct physical quantities, their perpendicular components exist in different dimensions, yet all are orthogonal to ***A***. The statistical assessment includes the endpoint ***A*** and the perpendicular to ***A*** components, namely ***B_y_*** and ***C_z_***. The ***B_x_*** and ***C_x_*** components are not utilized in the statistical analysis as they are parallel to ***A***, indicating complete similarity.

Considering clinical endpoints as vectors allows for the application of methods similar to those used in vector algebra. The VBC concept is graphically shown in Figure 3:

Certainly, in the rare case where there is only a single endpoint in a clinical study, there is no reason (and benefit) to apply the VBC methodology; however, in the vast majority of clinical studies, two or more endpoints are utilized. Using a clearly outlined process, the pairwise relationships between the remaining N-1 endpoints and E1 are estimated, then the N-1 endpoints are decomposed into axes orthogonal to E1. The angle formed between vectors offers an understanding of the degree of interdependence among variables. Vectors that are orthogonal to each other are regarded as independent, and solely these are used in the statistical analysis.

In the case of N clinical endpoints, the following steps are proposed:

Step 1: Define a Cartesian coordinate system. The coordinate system creates a relationship between vectors and their values in Euclidean space;

Step 2: Define the most important primary endpoint (denoted as E1). This would be the endpoint that holds the highest significance in the clinical study;

Step 3: Set the X-axis of the coordinate system to coincide with the axis of the most important endpoint (i.e., E1). Thus, every axis perpendicular to E1 would serve as the vertical axis of the coordinate system;

Step 4: Apply normalization, typically standardization, to all endpoints to make them unitless;

Step 5: Calculate the norm (magnitude) of each endpoint;

Step 6: Calculate the angle between each endpoint and the E1 endpoint. This estimation is based on the cosine similarity between each endpoint and E1;

Step 7: Decompose all endpoints into an X component and another component perpendicular to X. Plausibly, the E1 endpoint is by default the X axis, and no decomposition is made. All the remaining N-1 endpoints are decomposed into the X (e.g., B_x_, C_x_) and a perpendicular to X component (e.g., B_y_, C_z_);

Step 8: Perform the statistical analysis for E1, and each of the perpendicular projections of the remaining endpoints (e.g., B_y_, C_z_). This statistical analysis would be the one mandated by the regulatory authorities and is not affected by the vector decomposition used in the VBC approach.

**Two endpoints**

For the simple case of two endpoints/vectors (***A***, ***B***), the following route allows calculating the angle between the pair (***A***, ***B***) of endpoints (vectors).

1. Start by estimating the dot product between ***A*** and ***B***:

(1) $$A\cdot B=\sum_{i=1}^{K}\left(A_{i}\cdot B_{i}\right)=A_{1}\cdot B_{1}+A_{2}\cdot B_{2}+\cdots +A_{K}\cdot B_{K}$$

where *K* is the number of subjects participating in the trial;

2. Estimate the Euclidean norm (i.e., magnitude) for each vector (endpoint) ***A*** and ***B***:

(2) $$\left\|A\right\|=\sqrt{A_{1}^{2}+A_{2}^{2}+\ldots +A_{K}^{2}}$$

(3) $$\left\|B\right\|=\sqrt{B_{1}^{2}+B_{2}^{2}+\ldots +B_{K}^{2}}$$

3. From Equations (1) and (2), the angle (θ) between vectors ***A*** and ***B*** can be estimated:

(4) $$A\cdot B=\left\|A\right\|\left\|B\right\|cos\theta ⟺cos\theta =\frac{A\cdot B}{\left\|A\right\|\left\|B\right\|}\Rightarrow \theta ={cos}^{-1}\left(\theta \right)$$

4. Assuming that ***A*** is the most important endpoint (in line with Figure 3), endpoint ***B*** is decomposed onto the Y-axis. The angle θ is necessary to calculate this projection of ***B*** and estimate ***B_y_*** from Equation (5):

(5) $$B_{y}=B\cdot sin\left(\theta \right)$$

5. Finally, the appropriate statistical analysis is applied to endpoints ***A*** and ***B_y_***.

**More than two endpoints**

The abovementioned steps 1–4 can easily be generalized for the case of N endpoints.

It should be reminded that the cosine of the angle between vectors offers insight into the level of dependency between variables. A cosine value close to zero suggests that the vectors are almost orthogonal, implying independence between the random variables. Conversely, a cosine value near one or negative one indicates that the vectors are nearly parallel, indicating a strong correlation between the random variables [45].

In Section 2.3 and Section 2.4, the aforementioned process is further elaborated for the specific case of bioequivalence studies. In the “Discussion” section, further clarifications are provided regarding the concept of VBC.

### 2.3. Application of VBC: The Case of Bioequivalence Studies

**Figure 4:**
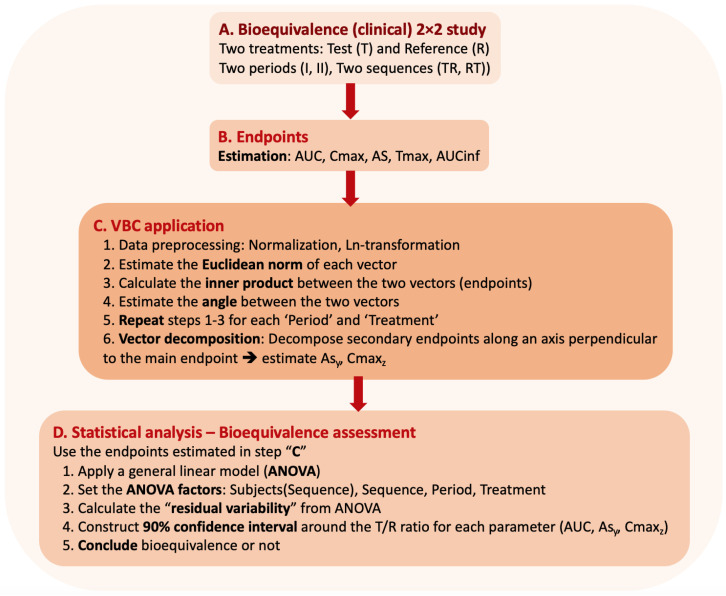
The most important steps in bioequivalence assessment. Stages (**A**,**B**,**D**) are mandated by the regulatory authorities. For VBC application, an extra step (**C**) is added to the entire procedure.

While Figure 3 shows the decomposition-based idea of VBC, Figure 4 depicts the entire procedure for a bioequivalence study. Certainly, similar steps can be followed for any clinical study, although the specific statistical framework may vary.

In the case of bioequivalence studies, two pharmaceutical products (test and reference) of the same active moiety and the same strength are compared. The comparison relies on the pharmacokinetic ground and two co-primary endpoints are compared according to the regulatory guidelines [23,24]. These two endpoints refer to the area under the concentration–time curve (AUC) from time zero until the last measured concentration, and the maximum observed plasma concentration (Cmax). The first term, AUC, refers to the extent of absorption, while Cmax is supposed to reflect the rate of absorption [23,24]; however, recent studies utilizing several machine learning algorithms showed that Cmax fails to characterize the absorption rate, while a newly proposed parameter, termed as the average slope (AS) succeeds in expressing absorption rate and showed clear superiority over Cmax [37,38,39]. Therefore, in this study, AS is further assessed using the VBC approach. Also, some pharmacokinetic parameters were used as surrogate endpoints. The latter includes the time (Tmax) at which Cmax appears and the AUC extrapolated to infinity (AUCinf) [23,24].

For the purposes of this study, plasma concentration–time (C-t) data of three cardiovascular drugs (amlodipine, irbesartan, and hydrochlorothiazide) were simulated using the model parameters reported in the literature [46,47,48,49,50,51,52,53,54,55]. The conditions of the typical 2 × 2 (two periods, two treatments, two sequences) crossover design were simulated (Figure 4A). A number of 200 subjects were simulated for each drug and 200 C-t profiles were generated for each case, which in a subsequent step were used to calculate the pharmacokinetic parameters under study (i.e., AUC, Cmax, AS, AUCinf, Tmax) using non-compartmental approaches, to be in line with the regulatory requirements (Figure 4B) [23,24].

If the typical bioequivalence procedure is to be followed, then the procedure should move to step D in Figure 4, which involves the statistical assessment; however, in the case of VBC, an intermediate step is added. In this step C, initially, normalization (specifically standardization) is applied to the individual pharmacokinetic parameters and then ln-transformation since it is required by the regulatory authorities in bioequivalence assessment (Figure 4C) [23,24]. Then, the vector decomposition steps are applied as described in Section 2.2. After the VBC step, the analysis continues as usual by performing the statistical assessment (Figure 4D) mandated by the regulatory authorities [23,24].

In more detail, the VBC stage involves all the steps presented in Section 2.2. In the example analyzed in this study, three endpoints are used: AUC, Cmax, and AS. The endpoint AUC is considered the most important; therefore, Cmax and AS are decomposed onto axes perpendicular to AUC. Since Cmax and AS are different parameters with different physical properties and units, they are decomposed into different dimensions.

The VBC route in the case of bioequivalence of three endpoints is outlined in the following steps:

i Estimation of the Euclidean norm of each vector (AUC, Cmax, and AS);
ii Calculation of the two inner products with respect to AUC (since it is considered the most important endpoint: $\langle AUC,Cmax\rangle$ and $\langle AUC,AS\rangle$;
iii Estimation of the angles: ∠(AUC·0·Cmax) and ∠(AUC·0·AS). Estimation of the angles is necessary since in a subsequent step the projections of Cmax and AS will be calculated using this angle;
iv Repeat steps “i–iii” for each Period and Treatment of the 2 × 2 study. Since our example refers to the typical case of bioequivalence studies, we use a 2 × 2 two period, two sequence, crossover clinical design;
v Perform vector decomposition onto the axes perpendicular to AUC. These perpendicular axes refer to Y and Z for AS and Cmax, respectively. In this step, we need the angles calculated in step “iii”. The decomposed endpoints are calculated in accordance with Equation (5), namely: AS_y_ = AS·sin(θ) and Cmax_z_ = Cmax·sin(φ), where θ and φ refer to the angles ∠(AUC·0·AS) and ∠(AUC·0·Cmax), respectively;
vi Proceed to the appropriate statistical analysis. In the case of bioequivalence assessment for three endpoints we need to compare: AUC_T_ vs. AUC_R_, AS_yT_ vs. AS_yR_, and Cmax_zT_ vs. Cmax_zR_, where T and R refer to the test and reference treatment, respectively.

The statistical analysis follows the official requirements of the regulatory guidelines [23,24]. This includes ln-transformation to all pharmacokinetic parameters and the application of analysis of variance (ANOVA) using the following factors: the nested term subject-within-sequence, treatment (T or R), sequence of administration (i.e., TR or RT), and period (first or second). From the ANOVA analysis, the residual variability was calculated, which was then used to construct the 90% confidence interval around the T/R ratio (actually the difference since all parameters are in the ln-domain) for each pharmacokinetic parameter (AUC, AS_y_, and Cmax_z_). Bioequivalence is declared if the 90% confidence interval lies between the acceptance limits of 80–125% [23,24].

### 2.4. Simulation Framework

The simulation methodology used in this study relied on the recently introduced in vitro–in vivo simulation (IVIVS) approach [56]. Nevertheless, substantial modifications were necessary, and additional components had to be incorporated: (a) estimating the AS in addition to the traditional pharmacokinetic endpoints, (b) conducting iterations for sequential ratios of the absorption rate constants for T and R product (KaT and KaR, respectively), and (c) recording the intermediate estimates of each iteration [57].

For each one of the three drugs (amlodipine, irbesartan, and hydrochlorothiazide), the proper models and parameter values were extracted from the literature (Table 1) [46,47,48,49,50,51,52,53,54,55]. A one-compartment model following first-order absorption and elimination was applied to analyze amlodipine, whereas irbesartan and hydrochlorothiazide kinetics were characterized by a two-compartment model incorporating a lag-time in absorption and first-order kinetic transfers. In the IVIVS procedure, these models were incorporated through ordinary differential equations by introducing suitably stochastic errors to each model parameter, accounting for between-subject variability, within-subject variability, and residual error [56,57].

The generation process proceeded with the simulation of virtual volunteers, using a lognormal statistical distribution. An unlimited number of individuals can be simulated, with these virtual subjects randomly assigned to one of the study’s two groups to receive either the T or R formulation. For the purposes of this study, a total of 200 virtual subjects were simulated for PCA, while for the Monte Carlo simulations, 24 subjects were generated for each 2 × 2 study. A proportional residual error model was used, while the between- and within-subject variability was set at 15% and 20%, respectively, for every model parameter. Appropriate sampling schemes fitted to the pharmacokinetic properties of each drug were used to select C-t data, which were then utilized to calculate pharmacokinetic metrics for each of the virtual patients [56,57].

**Figure A1:**
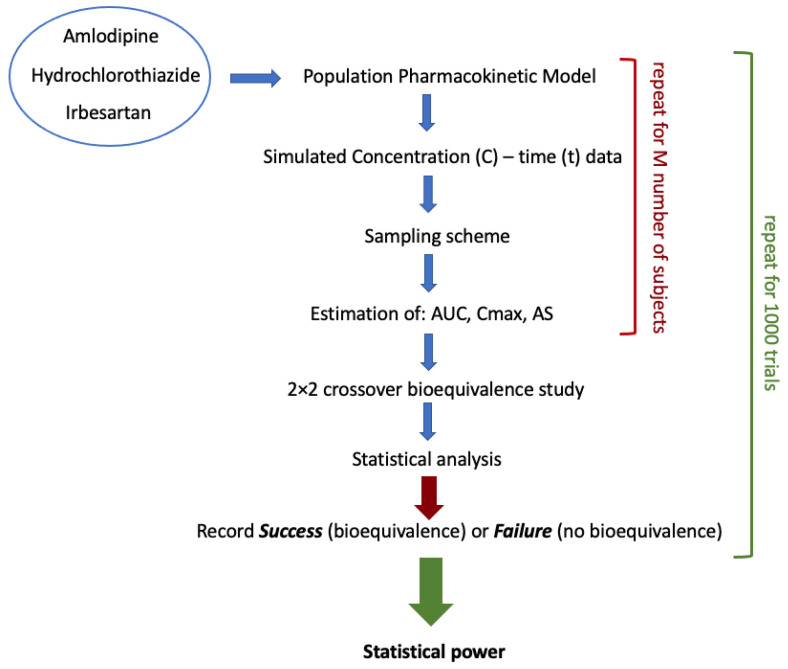
A graphical representation of the simulation methodology is presented. For each drug, an appropriate population pharmacokinetic model was developed, which was used to generate the concentration–time (C-t) data. To mimic real conditions, an appropriate sampling scheme was applied, and the three pharmacokinetic endpoints (AUC, Cmax, AS) were calculated. The process is repeated for M number of subjects, all data are organized in a 2 × 2 clinical design, and statistical analysis follows. The entire procedure is repeated 1000 times, and the success (bioequivalence) or failure (lack of bioequivalence) is recorded for each repetition, and, eventually, the statistical power is calculated.

A number of 1000 simulated 2 × 2 trials were generated for each scenario and condition (KaT/KaR ratio). The success or failure of each study, along with the geometric mean ratio (GMR) of each parameter (e.g., GMR_AUC_ for AUC), was determined. Moreover, the entire process was repeated for various KaT/KaR ratios starting from 1 (implying complete similarity in the average values of T and R), up to 2.0 (i.e., 100% discrepancy) with a step of 0.1. Following all repetitions, the % probability of BE acceptance and GMR estimates of all parameters were obtained as a function of KaT/KaR [56,57]. Additionally, the joint acceptances, where the two pharmacokinetic endpoints exhibit bioequivalence simultaneously, were recorded. The pairs of pharmacokinetic endpoints refer to (AUC, Cmax_z_) and (AUC, AS_y_). A graphical illustration of the simulation methodology is depicted in Figure A1.

The entire computational procedure was implemented in MATLAB^®^ R2024a (MathWorks, Natick, MA, USA) by developing the appropriate code and applying numerous validation measures [56,57].

### 2.5. Principal Component Analysis

Principal component analysis is an unsupervised machine learning method used to reduce the dimensionality of a high-dimensional feature set [42,43,44]. PCA creates a linear combination of the data dimensions to capture the maximum possible variability. The greatest fluctuations in the data occur along the direction of the first principal component, followed by the second dimension, and so on. “Loadings” indicate the contribution of each original variable to the new dimension. A feature’s impact on the principal component increases as the loading value approaches +1 or −1. The angle between a variable and a principal component illustrates how a feature contributes to the dominant component. Thus, loading plots offer insight into how strongly each attribute influences a significant component. The “biplot” is a common method for representing loadings and scores simultaneously [42,43,44]. It is a two-dimensional scatter plot with axes reflecting the two largest explained variance components. In this coordinate system, scores serve as coordinates for the loadings of the first two primary components of each feature. Scree plots help determine the number of important principal components, with the first component explaining the highest proportion of variability, the second explaining a moderate amount, and the third/fourth explaining only minor amounts. The entire PCA analysis was executed in Python v. 3.12.2.

## 3. Results

The aim of this study was to introduce a novel approach for evaluating clinical trials with multiple endpoints, considering not only their numerical values but also their physical properties and interrelationships. The newly proposed VBC approach allows for the discrimination and utilization only of the endpoint component expressing different attributes. VBC is inspired by dimension reduction and vector algebra principles and considers clinical endpoints as vectors. Using bioequivalence trials as a case study, the application of the VBC method is demonstrated to datasets from three cardiovascular drugs (amlodipine, irbesartan, and hydrochlorothiazide), each with distinct absorption characteristics.

### 3.1. Dimension Reduction Analysis

**Figure 5:**
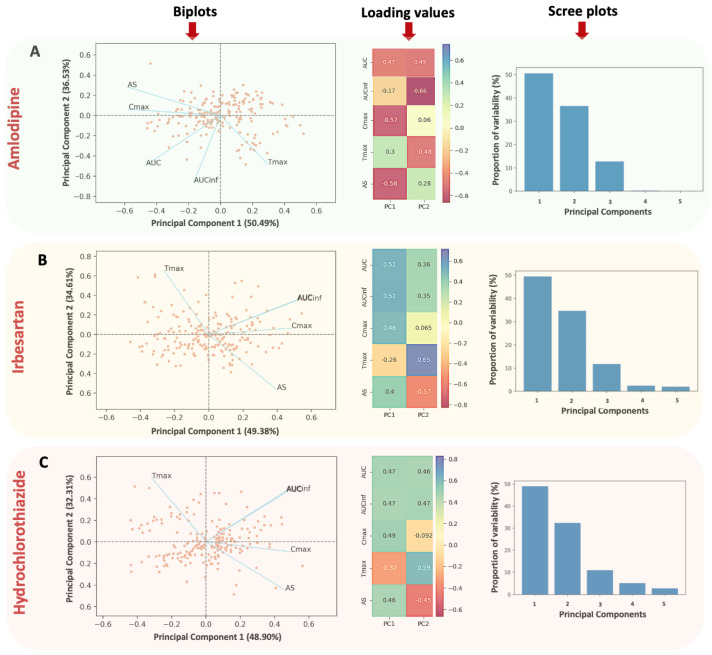
Biplots, loading values, and scree plots for the principal component analysis applied to amlodipine (**A**), irbesartan (**B**), hydrochlorothiazide (**C**). The pharmacokinetic endpoints assessed include the following: area under the curve up to the last quantifiable concentration (AUC), maximum observed plasma concentration of the drug (Cmax), the time Tmax at which Cmax occurs, AUC extrapolated to infinity (AUCinf), and average slope (AS).

Firstly, PCA was utilized to study the associations among the pharmacokinetic endpoints in a 5-dimensional space (since five pharmacokinetic parameters were used), of the three cardiovascular drugs with diverse absorption kinetics. The PCA results for amlodipine, irbesartan, and hydrochlorothiazide are depicted in Figure 5. The closed circles on the graph refer to the participants and the lines represent the variables vectors (AUC, AUCinf, Cmax, Tmax, and AS). The loadings of the 1st and 2nd principal components, for all pharmacokinetic endpoints, are presented next to the PCA graph.

Across all three drugs, a consistent pattern is noticeable; the vector of AS appears diametrically opposite to Tmax (with an angle close to 180°), indicating an inverse kinetic relationship. In simpler terms, while AS increases due to faster absorption, Tmax decreases (as it should be) and occurs at prior time points. Therefore, AS succeeds in reflecting the dynamic aspect of absorption. AUC and AUCinf are closely aligned and likely share similar loading values, particularly for irbesartan and hydrochlorothiazide (Figure 5B,C). Both AUC terms are nearly perpendicular to the Tmax axis, indicating a weak or even negligible relationship with the absorption rate.

The ideal situation for a metric to express absorption rate would be one where it lies diametrically opposite to Tmax. In all three plots of Figure 5, this is true only for AS but not for Cmax, which lies between the AUC and AS vectors. This implies that Cmax has a closer relationship with the extent of absorption (i.e., AUC and/or AUCinf) rather than Tmax. Certainly, Cmax is not entirely independent of the absorption rate like AUC but it does have a small relationship with the absorption rate; as depicted in Figure 5A–C, the angle between Cmax and AS is not 90 degrees (i.e., independent orthogonal vectors) but the angle is acute (see Figure 2). Similarly, the angle between Cmax and Tmax is obtuse (see Figure 2), and not 90 degrees indicating independence, or 180 degrees suggesting a completely negative relationship. All these findings imply that Cmax also significantly reflects the extent of absorption. These results are consistent across all three drugs, indicating the robustness of the associations among the pharmacokinetic endpoints.

The appropriateness of these PCA models is evident from their high descriptive ability (i.e., the % explained variance). For, amlodipine the first and second principal components account for 87.02% of total variability (50.49% and 36.53%, respectively). For irbesartan, the total explained variability is 83.99% (49.38% and 34.61%), while for hydrochlorothiazide it is 81.21% (48.90% and 32.31%). Scree plots were used to define the optimal number of components for the PCA models of amlodipine, irbesartan, and hydrochlorothiazide (Figure 5) using the criterion of identifying the “elbow” curve where the line flattens out.

### 3.2. Simulated Bioequivalence Studies

In order to investigate the performance of the VBC approach, Monte Carlo simulated bioequivalence studies were generated. In each trial, the VBC approach was utilized along with the officially required, by the regulatory authorities, statistical framework (Figure 4). For each drug, several ratios of the absorption rate constants were explored, and in each case, a number of 1000 simulated trials were performed to allow for obtaining robust estimates. The bioequivalence acceptance for each pharmacokinetic endpoint was recorded for each trial, and at the end, the overall percent acceptance for this endpoint was plotted against the absorption rate proportion (KaT/KaR). Not only the typical endpoints were assessed (AUC, Cmax, AS) but also the perpendicular-decomposed vectors Cmax_z_ and AS_y_.

#### 3.2.1. Individual Statistical Power

**Figure 6:**
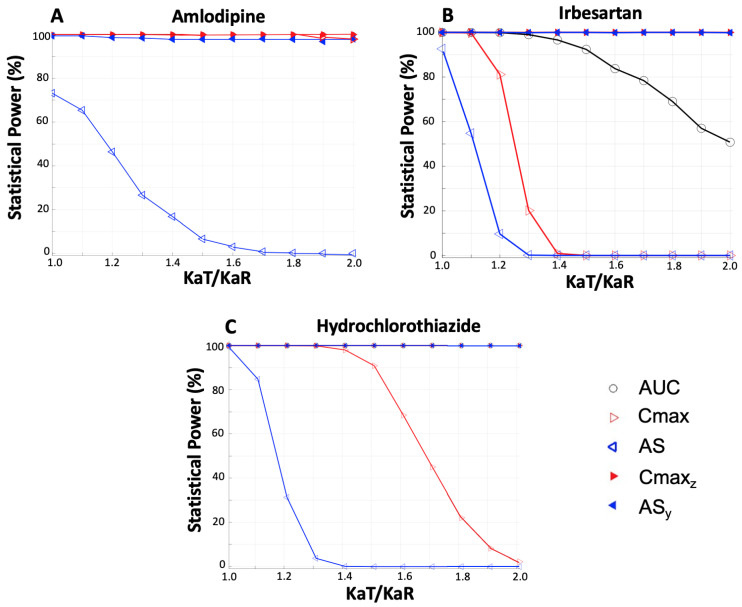
Statistical power plotted against the ratio of absorption rate constants between the test (KaT) and reference (KaR) pharmaceutical products. Three distinct plots are illustrated for each drug explored in this study: (**A**) Amlodipine, (**B**) Irbesartan, (**C**) Hydrochlorothiazide. Key: AUC, area under the concentration–time curve up to the last quantifiable concentration; Cmax, maximum observed plasma concentration of the drug; AS, average slope; Cmax_z_, the VBC-decomposed component of Cmax; AS_y_, the VBC-decomposed component of AS. The exact values are detailed in Table A1.

Figure 6 presents a summary of the statistical power outcomes for amlodipine (Figure 6A), irbesartan (Figure 6B), and hydrochlorothiazide (Figure 6C). Visual inspection of these plots reveals that AS exhibits the lowest probability of acceptance compared to all other endpoints. When Cmax is used as an endpoint, it shows a high probability of acceptance in the case of a moderately fast-absorbed drug like amlodipine (with Tmax around 5 h), while it shows lower % acceptances for hydrochlorothiazide (Tmax: 1–4 h), and similar to that of AS in the fast-absorbing case of irbesartan (Tmax: 1–2 h) [46,47,48,49,50,51,52,53,54,55]. It is noteworthy that in all other cases, very high acceptances were declared. The latter implies that mainly AS and to a lesser extent Cmax are accompanied by low statistical power. However, their VBC-decomposed counterparts overcome this drawback and can achieve high statistical power, namely, avoiding false-negative errors (i.e., type II errors). It should be stated that in Figure 6A Cmax cannot be clearly seen for KaT/KaR ratios up to 1.8 because it is almost superimposed with AUC and Cmax_z_; however, for higher KaT/KaR ratios, Cmax can be clearly observed since it gets lower. All results are detailed in Table A1 in Appendix A.

Additionally, a visual inspection of Figure 6 shows that the statistical power of AUC remains high (consistently at 100%) for all drugs and across all KaT/KaR ratios. This result is expected as AUC measures the extent of absorption, which is not affected by changes in the absorption rate constant. Also, as depicted earlier from the PCA analysis (Figure 5), AUC and AUCinf cannot express the rate of absorption, so the fact that they show high statistical power when assessing the absorption rate is useless. However, the observed high statistical power of AS_y_ is of paramount importance since it shows that the VBC method allows the use of an appropriate endpoint (i.e., AS) for expressing absorption rate but without its accompanied shortcomings, namely, the low statistical power.

#### 3.2.2. Joint Statistical Power

Figure 6 is useful to show that the VBC method allows using the most appropriate endpoint for expressing the desired characteristic, namely, the absorption rate in the case of bioequivalence studies; in addition, it shows that following the VBC steps (see Section 2.3), the statistical power is increased.

**Figure 7:**
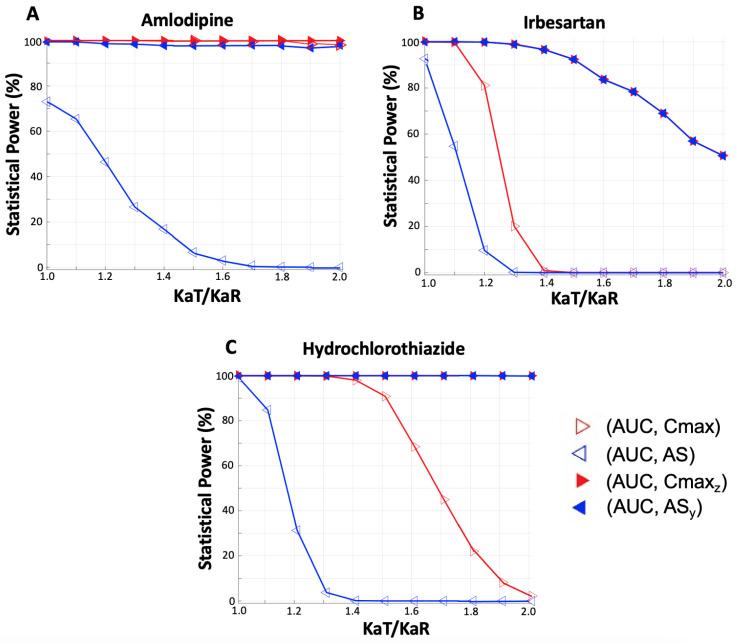
Joint statistical power plotted against the ratio of absorption rate constants between the test (KaT) and reference (KaR) pharmaceutical products. The joint acceptances refer to the situations where two pharmacokinetic endpoints exhibit bioequivalence simultaneously. The joint acceptances are illustrated for the pairs (extent, rate of absorption) of endpoints: (AUC, Cmax), (AUC, AS), (AUC, Cmax_z_), and (AUC, AS_y_). Three distinct plots are illustrated for each drug explored in this study: (**A**) Amlodipine, (**B**) Irbesartan, (**C**) Hydrochlorothiazide. Key: AUC, area under the concentration–time curve up to the last quantifiable concentration; Cmax, maximum observed plasma concentration of the drug; AS, average slope; Cmax_z_, the VBC-decomposed component of Cmax; AS_y_, the VBC-decomposed component of AS. The exact values are detailed in Table A2.

To further elaborate on these findings, Figure 7 was constructed, which presents the joint bioequivalence acceptance when both endpoints for the “extent” (AUC) and “rate” (Cmax or AS) of absorption are used. This situation mimics the actual practice in bioequivalence assessment since the regulatory authorities require that a study is successful when both co-primary endpoints pass the acceptance criteria [23,24]. Therefore, it depicts the joint acceptance for the pairs (extent, rate of absorption) of endpoints: (AUC, Cmax), (AUC, AS), (AUC, Cmax_z_), and (AUC, AS_y_).

Figure 7 reveals that the pairs of endpoints which utilize the pairs of AUC with the VBC-decomposed parameters, namely, (AUC, Cmax_z_) and (AUC, AS_y_), achieve very high acceptances (more than 90% or even close to 100%), while the traditional metrics exhibit much lower percentages, which become even lower as the KaT/KaR ratio deviates from unity.

In the case of a fast-absorbing drug (irbesartan, Figure 7B), the joint probability of acceptance for the pairs with the VBC-decomposed parameters starts to decrease only after a 40% difference in the absorption rate values, while for amlodipine and hydrochlorothiazide—which are absorbed much slower—the probability of acceptance remains almost 100% for KaT/KaR up to two. For example, in the case of irbesartan, a 30% difference in the Ka values results in a 0.1% and 20.1% probability of acceptance for the (AUC, AS) and (AUC, Cmax) pair, respectively. At the same time, the % acceptance for the VBC-decomposed pairs remains high, specifically at 98.9% and 99.0% for (AUC, AS_y_) and (AUC, Cmax_z_), respectively.

These observations verify the findings of Figure 6 and the fact that the VBC approach allows using the most appropriate endpoint without loss of statistical power. It should be noted that in Figure 7A, the (AUC, Cmax) pair is not clearly visible for KaT/KaR ratios up to 1.8 because it is nearly superimposed with that of (AUC, Cmax_z_); however, for higher KaT/KaR ratios, the % BE acceptance of the (AUC, Cmax) pair becomes clearly observable as it decreases. All results are detailed in Table A2 in Appendix A.

## 4. Discussion

Multiple endpoints are crucial in clinical studies [9,11,12]; however, multiplicity problems can arise in clinical studies when assessing multiple endpoints, posing challenges in data analysis and interpretation [18,19,20]. The more endpoints evaluated, the higher the probability of observing statistically significant results by chance alone, leading to inflated type I error rates. This phenomenon, known as the “multiplicity problem,” increases the risk of false-positive findings and can undermine the reliability of study conclusions. Multiplicity adjustments, such as Bonferroni’s correction or false discovery rate correction, are applied to control the familywise error rate or the false discovery rate in the context of multiple hypothesis testing [15,16,17]. Nevertheless, adjusting for multiple comparisons to control the overall type I error rate often requires stringent correction methods, like those outlined above, which may reduce statistical power and increase the likelihood of type II errors (i.e., false negatives). Reverse multiplicity is a term used to describe the situation where the statistical tests are performed in a way that increases the probability of false-negative conclusions [58,59,60,61]. This can occur when the tests are not properly adjusted for multiplicity, leading to a higher chance of missing a true positive result.

To address these issues, the VBC approach is introduced in this study. The concept of VBC originates from the recognition that endpoints in clinical trials can be viewed not only as measurable quantities but also as vectors. This perspective enables the utilization of mathematical tools from other scientific disciplines, such as vector algebra and PCA [40,41,44]. In vector algebra, vectors that align closely exhibit smaller angles between them, signifying greater similarity; conversely, vectors with larger angular separations indicate greater dissimilarity. Perpendicular vectors are considered independent, as changes in one component do not affect the other. Sets containing mutually perpendicular vectors are deemed independent sets. These characteristics align with terminology in dimension reduction techniques within machine learning. In the same context, PCA treats perpendicular vectors as independent. However, the practical application of PCA is limited due to its complexity and challenges in ensuring reproducibility. In contrast, vector algebra methods were applied through a simple and specific procedure (see Section 2.2).

**Figure 8:**
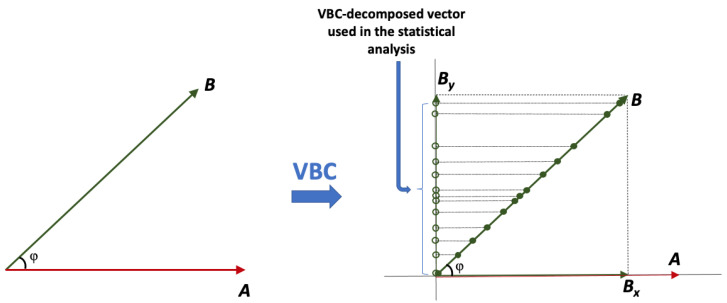
A graphical illustration of vector decomposition according to the VBC theory considers endpoint ***A*** as the most important, while endpoint ***B*** is projected onto the Y-axis to form the ***B_y_*** component, which is orthogonal (independent) to ***A***. This projection transforms all ***B*** values (filled circles) into their ***B***·sin(φ) equivalents (open circles). Crucially, this process is a linear operation, preserving the shape of the distribution of the actual data. As all data are multiplied by sin(φ), which never exceeds 1, the resulting ***B_y_*** values are compressed, resulting in reduced variability and increased statistical power compared to the original. Overall, the proposed VBC approach achieves two objectives: (a) utilizing solely the unrelated part of the vectors, and (b) reducing the variability of the decomposed endpoints.

Apart from enabling the consideration of similarity between primary endpoints, the VBC approach also increases the statistical power of the study. This attribute arises from applying vector decomposition to isolate the independent component of the vector, particularly concerning the most crucial primary endpoint (i.e., E1). Figure 8 provides a graphical representation illustrating how the VBC achieves an increase in statistical power. The original endpoint values are depicted as dots along the vector dimension (i.e., ***B***). Upon applying vector decomposition to calculate the ***B_y_*** component, these dots are projected onto the Y-axis. This projection requires transforming all ***B*** values into their **B**·sin(φ) equivalents. It is important to note that the projection is a linear operation, thus preserving the shape of the distribution of the actual data; however, as the data are projected onto the Y-axis and multiplied by sin(φ), which is always less than or equal to 1, the ***B_y_*** values are compressed, resulting in reduced variability compared to the original. In turn, this reduction in variability contributes to an increased statistical power. Overall, the proposed VBC approach, as discussed in Section 2.2.3 (and Section 2.3 concerning BE studies), achieves two objectives: (a) utilization of solely the unrelated part of the vectors in comparison to the predetermined most important endpoint, and (b) reduction in the variability of the decomposed endpoints, consequently leading to an increase in statistical power. It is noteworthy that the projection in vector decomposition is a linear operation, thereby preserving the shape of the original distribution.

The problem that the proposed VBC approach aims to address, lies in the complexity that arises when needing to assess multiple characteristics; the latter raises the need of using multiple endpoints, which subsequently pose several challenges [18,19,20]. Firstly, these endpoints may be interrelated, rendering some redundant or partially redundant, thereby questioning their utility. Secondly, the need to adjust for multiplicity to control type I error rates adds complexity, and such adjustments can increase the risk of type II errors, thus, reducing the sensitivity of the analysis. To overcome these issues, the VBC approach allows for the discrimination and utilization only of the endpoint component expressing different attributes. This permits the assessment of the (dis)similarity among them, enabling the utilization of only the unrelated components of the endpoints. Furthermore, the stepwise procedure outlined in Section 2.2 and Section 2.3 has an additional advantage as it reduces the variability of each endpoint and increases the statistical power in this study.

Table 2 outlines the main aspects of the VBC approach introduced in this study.

Bioequivalence assessment is a typical example where multiple co-primary endpoints are individually tested, each at a nominal significance level (5%) [62,63,64]; therefore, in this study, VBC is applied to the bioequivalence analysis of three drugs used commonly in cardiology. While the same procedures can be adapted for various clinical studies, the statistical framework will vary accordingly.

These three drugs were chosen based on their distinct absorption kinetics: irbesartan exhibits the fastest absorption, reaching its Tmax (time to peak concentration) within 1–2 h after administration; hydrochlorothiazide shows moderate absorption, with a Tmax of up to 4 h; whereas amlodipine has the slowest absorption, with a Tmax of 5 h following oral administration. In addition to the standard bioequivalence endpoints mandated by regulatory agencies, such as AUC and Cmax, this study also incorporated the use of newly introduced metrics, including AS, as well as Tmax and AUCinf. AS, which stands for average slope, demonstrated favorable properties in reflecting absorption rate and was suggested as a potential alternative to address issues associated with Cmax [37,38,39]. Based on the literature-derived pharmacokinetic model parameters, simulated concentration–time data were generated, and thereafter, the corresponding pharmacokinetic endpoints were computed [49,50,51,52,53,54,55,56,57]. To achieve this, 200 virtual subjects were generated.

Initially, PCA was initially applied to examine the underlying relationships among the pharmacokinetic endpoints of the three drugs. For all three PCA analyses, the high percentages of explained variance (>80%) demonstrate the anticipated descriptive capability of the PCA models developed. PCA results indicated similar performance for all three drugs. AS, which is adjacent and inversely proportional to Tmax, denotes the inverse kinetic behavior of AS and Tmax. While AS increases, indicating faster absorption, Tmax decreases, occurring at earlier time points, effectively depicting the dynamic aspect of absorption. AUC and AUCinf are positioned almost perpendicularly to the AS-Tmax direction, highlighting the weak link between AUC and absorption rate. Cmax lies between the AS and AUC vectors, indicating a weak relationship with the absorption rate. Despite differences in absolute positions, the relative positions among the pharmacokinetic vectors are similar, attributed to the use of drugs with varying absorption kinetics and rotation settings in PCA analysis. Results confirm that Cmax is only slightly related to absorption rate, while AS closely reflects it.

Consequently, PCA demonstrated the necessity of using the VBC approach, as all endpoints exhibited varying degrees of relevance. The VBC approach is a simplification of PCA that can be easily used straightforwardly in practice and provide reproducible results. VBC enables the utilization of each endpoint informative component in addition to what was already expressed by another endpoint. Given the indisputable use of AUC for reflecting the extent of absorption, there was a need to express the true rate of absorption. Thus, AUC was considered the most important endpoint (referred to as “E1” in Section 2.2), and all other endpoints were decomposed relative to AUC. The AS component perpendicular to AUC (i.e., AS_y_) was utilized. For comparative purposes, the Cmax_z_ component was also utilized, even though PCA indicated that Cmax expresses both the extent and rate of absorption, not just the rate of absorption as intended [37,38,39].

Following the VBC approach and using the endpoints mentioned above, Monte Carlo simulations of bioequivalence studies were performed [56,57]. The aim of this part of the study was to explore the performance of the VBC-decomposed endpoints (AS_y_, Cmax_z_), in terms of the statistical power in comparison with the conventional endpoints (i.e., AUC, Cmax). Also, to mimic real conditions where bioequivalence is declared when it is proved both for the two co-primary endpoints, joint bioequivalence acceptances were also recorded. For all three drugs, the simulations (Figure 6) showed that AS_y_ demonstrates high statistical power, indicating the effectiveness of the VBC method in utilizing an appropriate endpoint (i.e., AS) to express absorption rate without the associated shortcomings, such as increased type II error. In the same vein, simulations using the joint acceptances (Figure 7) further confirmed that the VBC approach enables the utilization of the most appropriate endpoint without sacrificing statistical power.

Another important aspect is the rationale behind selecting the normalization method for the endpoints. Initially, it is important to note that normalization is necessary because the endpoints represent different physical quantities measured in varying units and scales. In the VBC approach, standardization was chosen among other methods, such as min–max scaling, range method, and robust scaler. Standardization, typically achieved through normalization, always maintains the shape of a variable distribution [65]. When you standardize a variable, its distribution shape remains unchanged. This process involves transforming the variable to have a mean of zero and a standard deviation of one by subtracting the mean from each value and then dividing by the standard deviation. While standardization adjusts the scale of the variable, it does not affect the relative distances between data points or the distribution shape. Whether the original variable follows a normal distribution or exhibits skewness, the standardized variable retains the same distribution characteristics. In essence, standardization modifies the scale of the variable while preserving its distribution shape.

A technical aspect of the VBC is the utilization of the Euclidean norm in this study. While other types of norms, such as the Taxicab (or Manhattan) norm or maximum norm, also exist, the Euclidean norm was chosen due to its straightforward geometric interpretation and its agreement with the Pythagorean theorem, both of which are crucial for our purpose [40,41].

To this point, it should be mentioned that this work is one of the studies that aim to increase the statistical power of clinical trials. Similar efforts by scientists in the field have also appeared in the literature. For example, in a recent study, the problem of clinical trial design is reformulated as an optimization challenge, integrating high-dimensional aspects [66]. In that study, the authors introduced a computational approach using Monte Carlo and smoothing techniques to address it. Their method uses modern techniques of general-purpose computing on graphics processing units for large-scale parallel computing [66]. In another quite recent study, the endpoints of Phase 2 and Phase 3 trials are examined within a combined 2-in-1 design [67]. In addition, a comprehensive introduction to the concept of a 2-in-1 design and its diverse applications are highlighted in the study of Chen and Zhang [68]. All of these applications entail the consideration of correlated multiple endpoints, providing a nuanced understanding of their implications and utility across various domains.

In this study, the VBC theory was applied to bioequivalence assessments since these kinds of studies require the assessment of two co-primary endpoints that are inherently related to each other; however, it should be highlighted that VBC can be used for any clinical study with multiple endpoints or any other situation/application involving comparisons across multiple interrelated characteristics, whether they are measured on a numerical or Likert scale. Examples may include weather forecasting (e.g., temperature, humidity), economic indicators (e.g., gross domestic product, inflation rate), and traffic flow variables (e.g., vehicle speed, traffic volume, or congestion levels), among others. The application of VBC in problems like those mentioned previously will enable the utilization of all endpoints, thereby capturing various aspects of the studied situation. Simultaneously, it allows avoiding drawbacks such as redundancy, increased type I and II errors, and the risk of overinterpretation. VBC offers also several other advantages, such as its reliance on a physical rationale. VBC, as a simplification of dimension reduction methods originating from vector algebra, considers endpoints as vectors, enabling their unrelatedness through vector decomposition and thus avoiding the use of redundant endpoints. While there are several methods available for orthogonalizing vectors or finding orthogonal bases for vector spaces, such as the Gram–Schmidt process, QR decomposition, or Householder transformation, the VBC method is preferred for its simplicity [41]. It can be easily applied in practice without the need for sophisticated software or background knowledge, and it is entirely reproducible.

A limitation of this study relies on the fact that simulated concentration–time data of the three drugs were used. Even though these simulations were derived from validated models and the literature information, it is necessary to apply VBC to actual (experimental) data. Additionally, due to computational workload, the number of Monte Carlo iterations within each scenario was limited to 1000. This is the underlying reason why small discrepancies are observed in the statistical power plots (e.g., Figure 7A,B). A higher number of iterations would lead to more robust estimates. Also, putting this new concept into practice would necessitate regulatory agencies establishing precise criteria and guidelines regarding its application to prevent potential pitfalls and ensure reproducibility. While this study has introduced the concept, subsequent testing across various domains, including clinical studies and beyond, will determine the practical implementation of the VBC approach.

This study outlines the entire process for a bioequivalence study, with potential applicability to various clinical studies, albeit with differing statistical frameworks. Future applications of VBC in bioequivalence could include highly variable drugs and modified-release products. In the case of high variability, which often leads to reduced statistical power, VBC could serve as a tool to address this issue without requiring additional subject recruitment. Additionally, the assessment of modified-release products often involves evaluating multiple endpoints simultaneously, which can increase the risk of multiplicity and reduce power. Clinical trials with multiple endpoints also present opportunities for VBC application. Nevertheless, VBC can be applied in everyday cases that involve comparing two or more characteristics.

## 5. Conclusions

The aim of this study was to introduce a novel approach, named VBC, for evaluating clinical trials with multiple endpoints. Traditional analysis of clinical trials does not consider the possible similarity among the measured endpoints, which can result in multiplicity issues, reduced statistical power, and the need to recruit more subjects. The VBC approach applies vector algebra principles and considers clinical endpoints as vectors. In this study, the VBC idea was initially described and then applied to the bioequivalence assessment of three antihypertensive drugs (amlodipine, irbesartan, and hydrochlorothiazide) with different pharmacokinetic properties. The results suggest that VBC is a reproducible, easily applicable method allowing for the discrimination and utilization of the endpoint component expressing different attributes. VBC also offers several other advantages such as its reliance on a physical rationale, avoiding the risk of endpoint redundancy and over-interpretation, as well as the assessment of all clinical characteristics with increased statistical power and no inflation of type I error. Additionally, the combined application of VBC with the recently proposed parameter AS for expressing absorption rate showed high statistical power. Finally, it should be noted that VBC can be used for any clinical study with multiple endpoints or any other situation involving comparisons across multiple interrelated characteristics. The application of VBC is rather simple, requiring neither scientific expertise nor sophisticated software. It can be utilized across various occasions, accommodating multiple endpoints or characteristics.

## Figures and Tables

**Table 1:** Key aspects of the computational part of this study.

Factors	Description
Medicines	Amlodipine Irbesartan Hydrochlorothiazide
PCA	
Sample size	200
Software	Python 3.12.2
Settings	Z-score standardization
Monte Carlo simulations	
Sample size	24
Between-subject variability	15%
Within-subject variability	20%
Software	MATLAB 2024a
Pharmacokinetic information	References
Amlodipine	[46,47,48]
Irbesartan	[49,50,51]
Hydrochlorothiazide	[52,53,54,55]
Simulations methodology	[56,57]

**Table 2:** An overview of the critical elements of this study.

Issue	Action Taken in This Study/Description
Multiple endpoints: - Related endpoints - Multiplicity - Decrease in statistical power	Introduce Vector Based Comparison (VBC)
Description of the VBC approach	Section 2.2 (general) Section 2.3 (specifically for bioequivalence studies)
Application of the VBC in this study	Bioequivalence studies of amlodipine, irbesartan, hydrochlorothiazide
Bioequivalence data to apply the VBC	Monte Carlo simulated bioequivalence studies
Endpoints used	AUC, AUCinf, Cmax, Tmax, AS
Identify relationships among the endpoints	Principal Component Analysis was applied to separate datasets of the three drugs
Fields of VBC application	Clinical studies Bioequivalence studies Any statistical comparison that includes two or more endpoints (characteristics)
Prerequisites of VBC	Any variables being on numerical or Likert scale
Advantages of VBC	Physical rationale Avoid using unnecessary (related) endpoints Avoid multiplicity issues Increase statistical power Reduce sample size Simple Reproducible

## Data Availability

The raw data supporting the conclusions of this article will be made available by the author upon request.

## Appendix A

**Table A1:** Statistical power as a function of the absorption rate constants ratio between the test (KaT) and reference (KaR) pharmaceutical products. The results are shown for each individual drug: (A) Amlodipine, (B) Irbesartan, (C) Hydrochlorothiazide.

**KaT/KaR**	**A. Amlodipine**				
	**AUC**	**Cmax**	**AS**	**Cmax_z_**	**AS_y_**
1.0	100.0	100.0	73.2	100.0	99.4
1.1	100.0	100.0	65.4	100.0	99.4
1.2	100.0	100.0	46.4	100.0	98.6
1.3	100.0	100.0	26.6	100.0	98.4
1.4	100.0	99.8	16.8	100.0	98.0
1.5	100.0	99.8	6.6	100.0	98.0
1.6	100.0	99.6	3.0	100.0	97.8
1.7	100.0	99.6	0.8	100.0	97.8
1.8	100.0	99.4	0.2	100.0	97.4
1.9	100.0	98.6	0.0	100.0	96.8
2.0	100.0	98.2	0.0	100.0	96.8
**KaT/KaR**	**B. Irbesartan**				
	**AUC**	**Cmax**	**AS**	**Cmax_z_**	**AS_y_**
1.0	100.0	100.0	92.6	100.0	100.0
1.1	100.0	99.8	54.8	100.0	100.0
1.2	99.9	81.1	9.6	100.0	100.0
1.3	99.0	20.1	0.1	100.0	100.0
1.4	96.6	0.9	0.0	100.0	100.0
1.5	92.4	0.0	0.0	100.0	100.0
1.6	83.8	0.0	0.0	100.0	100.0
1.7	78.4	0.0	0.0	100.0	100.0
1.8	69.0	0.0	0.0	100.0	100.0
1.9	57.0	0.0	0.0	100.0	100.0
2.0	50.8	0.0	0.0	100.0	99.9
**KaT/KaR**	**C. Hydrochlorothiazide**				
	**AUC**	**Cmax**	**AS**	**Cmax_z_**	**AS_y_**
1.0	100.0	100.0	99.3	100.0	100.0
1.1	100.0	100.0	84.8	100.0	100.0
1.2	100.0	100.0	31.3	100.0	100.0
1.3	100.0	99.9	3.8	100.0	100.0
1.4	100.0	98.0	0.1	100.0	100.0
1.5	100.0	90.9	0.0	100.0	100.0
1.6	100.0	68.5	0.0	100.0	100.0
1.7	100.0	45.0	0.0	100.0	100.0
1.8	100.0	22.0	0.0	100.0	100.0
1.9	100.0	8.3	0.0	100.0	100.0
2.0	100.0	2.3	0.0	100.0	99.9

Key: AUC, area under the concentration–time curve up to the last quantifiable concentration; Cmax, maximum observed plasma concentration of the drug; AS, average slope; Cmax_z_, the VBC-decomposed component of Cmax; AS_y_, the VBC-decomposed component of AS.

**Table A2:** Joint statistical power as a function of the absorption rate constants ratio between the test (KaT) and reference (KaR) pharmaceutical products. The results are shown for each individual drug: (A) Amlodipine, (B) Irbesartan, (C) Hydrochlorothiazide. The joint acceptances refer to situations where two pharmacokinetic endpoints exhibit equivalence simultaneously. The joint acceptances are illustrated for the pairs (extent, rate of absorption) of endpoints: (AUC, Cmax), (AUC, AS), (AUC, Cmax_z_), and (AUC, AS_y_).

**KaT/KaR**	**A. Amlodipine**			
	**(AUC, Cmax)**	**(AUC, AS)**	**(AUC, Cmaxz)**	**(AUC, ASy)**
1.0	100.0	73.2	100.0	99.4
1.1	100.0	65.4	100.0	99.4
1.2	100.0	46.4	100.0	98.6
1.3	100.0	26.6	100.0	98.4
1.4	99.8	16.8	100.0	97.8
1.5	99.8	6.6	100.0	97.8
1.6	99.8	3.0	100.0	98.0
1.7	99.4	0.8	100.0	98.0
1.8	99.4	0.2	100.0	97.8
1.9	98.6	0.0	100.0	96.8
2.0	98.2	0.0	100.0	98.0
**KaT/KaR**	**B. Irbesartan**			
	**(AUC, Cmax)**	**(AUC, AS)**	**(AUC, Cmaxz)**	**(AUC, ASy)**
1.0	100.0	92.6	100.0	100.0
1.1	99.8	54.8	100.0	100.0
1.2	81.1	9.6	99.9	99.9
1.3	20.1	0.1	99.0	98.9
1.4	0.9	0.0	96.6	96.6
1.5	0.0	0.0	92.4	92.4
1.6	0.0	0.0	83.8	83.6
1.7	0.0	0.0	78.4	78.4
1.8	0.0	0.0	69.0	69.0
1.9	0.0	0.0	57.0	57.0
2.0	0.0	0.0	50.8	50.6
**KaT/KaR**	**C. Hydrochlorothiazide**			
	**(AUC, Cmax)**	**(AUC, AS)**	**(AUC, Cmaxz)**	**(AUC, ASy)**
1.0	100.0	99.3	100.0	100.0
1.1	100.0	84.8	100.0	100.0
1.2	100.0	31.3	100.0	100.0
1.3	99.9	3.8	100.0	100.0
1.4	98.0	0.1	100.0	100.0
1.5	90.9	0.0	100.0	100.0
1.6	68.5	0.0	100.0	100.0
1.7	45.0	0.0	100.0	100.0
1.8	22.0	0.0	100.0	100.0
1.9	8.3	0.0	100.0	100.0
2.0	2.3	0.0	100.0	100.0

Key: AUC, area under the concentration–time curve up to the last quantifiable concentration; Cmax, maximum observed plasma concentration of the drug; AS, average slope; Cmax_z_, the VBC-decomposed component of Cmax; AS_y_, the VBC-decomposed component of AS.
